# Intrathecal delivery of PDGF produces tactile allodynia through its receptors in spinal microglia

**DOI:** 10.1186/1744-8069-5-23

**Published:** 2009-05-11

**Authors:** Junya Masuda, Makoto Tsuda, Hidetoshi Tozaki-Saitoh, Kazuhide Inoue

**Affiliations:** 1 Department of Molecular and System Pharmacology, Graduate School of Pharmaceutical Sciences, Kyushu University, 3-1-1 Maidashi, Higashi-ku, Fukuoka, Fukuoka 812-8582, Japan

## Abstract

Neuropathic pain is a debilitating pain condition that occurs after nerve damage. Such pain is considered to be a reflection of the aberrant excitability of dorsal horn neurons. Emerging lines of evidence indicate that spinal microglia play a crucial role in neuronal excitability and the pathogenesis of neuropathic pain, but the mechanisms underlying neuron-microglia communications in the dorsal horn remain to be fully elucidated. A recent study has demonstrated that platelet-derived growth factor (PDGF) expressed in dorsal horn neurons contributes to neuropathic pain after nerve injury, yet how PDGF produces pain hypersensitivity remains unknown. Here we report an involvement of spinal microglia in PDGF-induced tactile allodynia. A single intrathecal delivery of PDGF B-chain homodimer (PDGF-BB) to naive rats produced a robust and long-lasting decrease in paw withdrawal threshold in a dose-dependent manner. Following PDGF administration, the immunofluorescence for phosphorylated PDGF β-receptor (p-PDGFRβ), an activated form, was markedly increased in the spinal dorsal horn. Interestingly, almost all p-PDGFRβ-positive cells were double-labeled with an antibody for the microglia marker OX-42, but not with antibodies for other markers of neurons, astrocytes and oligodendrocytes. PDGF-stimulated microglia *in vivo *transformed into a modest activated state in terms of their cell number and morphology. Furthermore, PDGF-BB-induced tactile allodynia was prevented by a daily intrathecal administration of minocycline, which is known to inhibit microglia activation. Moreover, in rats with an injury to the fifth lumbar spinal nerve (an animal model of neuropathic pain), the immunofluorescence for p-PDGFRβ was markedly enhanced exclusively in microglia in the ipsilateral dorsal horn. Together, our findings suggest that spinal microglia critically contribute to PDGF-induced tactile allodynia, and it is also assumed that microglial PDGF signaling may have a role in the pathogenesis of neuropathic pain.

## Findings

Peripheral nerve damage leads to a persistent neuropathic pain state in which innocuous stimuli elicit pain behavior (tactile allodynia) [1-3]. Neuropathic pain might involve aberrant excitability of the nervous system, notably at the levels of the primary sensory ganglia and the dorsal horn of the spinal cord [4-8]. There is a rapidly growing body of evidence indicating that peripheral nerve damage activates glial cells in the dorsal horn and results in changing expression and activity of various molecules [9,10]. Importantly, pharmacological, molecular and genetic manipulations of the function or expression of glial molecules have been shown to substantially influence nerve injury-induced pain behaviors and hyperexcitability of the dorsal horn pain pathway [11-15]. Therefore, signaling between neurons and glia might critically contribute to the pathologically enhanced pain processing in the dorsal horn that underlies neuropathic pain. However, the mechanisms underlying neuropathic pain caused by neuron-glia communications in the dorsal horn remain to be fully elucidated.

Platelet-derived growth factors (PDGFs) and their receptors (PDGFRs) have served as prototypes for growth factor and receptor tyrosine kinase (RTK) function. The biologically active form of PDGF is a disulfide-bonded dimer of A-, B-, C-, or D-polypeptide chains. The PDGF isoforms (PDGF-AA, -AB, -BB, -CC, or -DD) bind two structurally related RTKs (PDGFRα and β). PDGF-AA, -BB, -AB, and -CC bind to PDGFRα, whereas PDGF-BB and -DD bind to PDGFRβ [16-20]. Ligand binding induces receptor dimerization and autophosphorylation, subsequently initiates downstream signaling, and causes cellular responses such as proliferation, differentiation, survival, migration, chemotaxis, and gene expression [21,22].

Although PDGF signaling is commonly known to have essential roles during development [23], there is limited evidence for its role in the mature CNS. A recent study has shown that PDGF is expressed in dorsal horn neurons in adult mice, and that intrathecal administration of either a selective inhibitor of PDGFR phosphorylation or an antibody trapping endogenous PDGF suppresses thermal hyperalgesia and tactile allodynia after peripheral nerve injury [24]. Thus, PDGF released from dorsal horn neurons is implicated in neuropathic pain. However, how PDGF produces pain hypersensitivity remains unknown.

**Figure 1:**
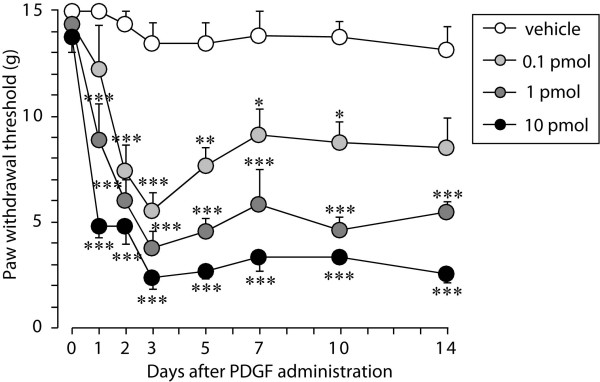
**A single intrathecal PDGF-BB administration produces tactile allodynia**. The paw withdrawal thresholds in response to mechanical stimuli were measured in rats intrathecally administered vehicle (n = 5) or PDGF-BB (0.1, 1 and 10 pmol, n = 5) just before administration (day 0) and 1, 2, 3, 5, 7, 10, and 14 days after administration. Data represent the means ± SEM of the thresholds. ***P < 0.001, **P < 0.01, *P < 0.05 vs vehicle group by repeated measures two-way ANOVA with Bonferroni post-hoc tests.

To investigate this, we first examined whether the intrathecal delivery of PDGF produces tactile allodynia in adult naive rats. We used the PDGF-BB isoform in all experiments in this study because PDGF-B chain expression is induced after peripheral nerve injury [25], neurons throughout the CNS contain the PDGF-B chain [26], and the PDGF-B chain activates both PDGFRα and PDGFRβ [21,23]. We found that a single intrathecal administration of PDGF-BB (0.1, 1 and 10 pmol) produced marked and long-lasting tactile allodynia: the paw withdrawal threshold in response to mechanical stimulation applied to the hindpaw progressively decreased over the first 3 days, reaching the lowest in the threshold on day 3, and this decrease persisted at least for 14 days after PDGF-BB administration (*P *< 0.001) (Figure 1). The PDGF-BB-induced tactile allodynia was dose dependent (Figure 1). In addition, we also tested the effect of AG17, a selective inhibitor for PDGFR phosphorylation, on PDGF-BB-induced allodynia. Consistent with the previous results in mice [24], intrathecal pretreatment with AG17 (100 nmol) significantly attenuated the decrease in the paw withdrawal threshold 7 days after PDGF administration (PDGF-BB + vehicle group, 1.71 ± 0.41, n = 4; PDGF-BB + AG17 group, 9.43 ± 1.13, n = 4; *P *< 0.001).

**Figure 2:**
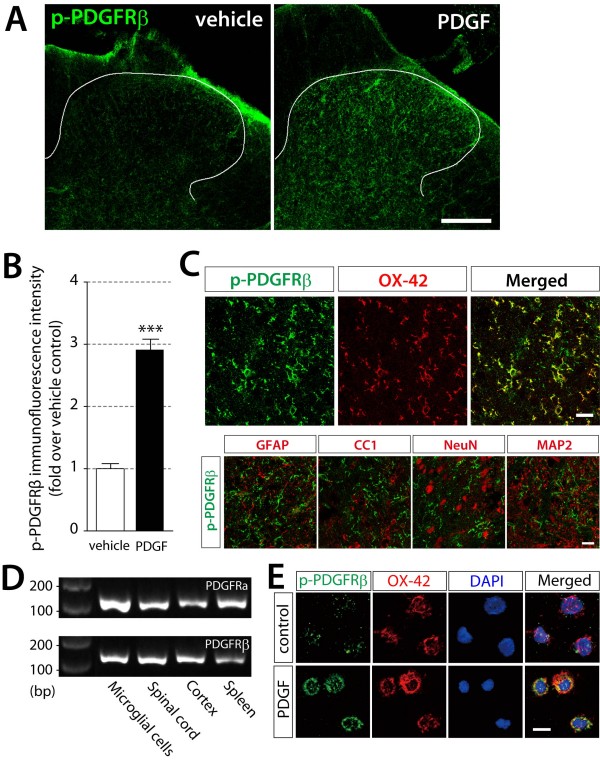
**PDGF-BB phosphorylates its receptors in spinalmicroglia**. (A) The immunoreactivity of phosphorylated PDGFRβ protein was detected by a specific antibody for p-PDGFRβ 30 min after intrathecal administration of vehicle or PDGF-BB (10 pmol) in the L5 spinal dorsal horn. Scale bar, 200 μm. (B) The intensity of p-PDGFRβ immunofluorescence was quantified in the dorsal horn region of vehicle treated rats and PDGF-BB treated rats. Data represent the means ± SEM of the immunofluorescence intensity (n = 5). ***P < 0.001 vs vehicle by Student's *t*-test. (C) Double immunofluorescence labeling of the dorsal horn 30 min after intrathecal PDGF-BB administration with p-PDGFRβ (green) and cell markers (red); OX-42, a microglia marker; GFAP, an astrocytes marker; CC1, an oligodendrocytes marker; NeuN and MAP2, neurons markers. Scale bars, 20 μm. (D) PDGFRα (116 bp) and PDGFRβ (145 bp) mRNA expression in primary microglia by RT-PCR analysis. Spinal cord, cerebral cortex, and spleen are positive controls. (E) Triple immunofluorescence labeling of p-PDGFRβ (green) with OX-42 (red) and DAPI (blue), a nuclear marker, in primary microglia treated with PBS as a control or PDGF-BB (50 ng/ml) for 10 min. Scale bar, 20 μm.

To identify the cell types on which intrathecally delivered PDGF-BB acts, we performed immunohistochemical experiments using an anti-phospho-Tyr1021 PDGFRβ antibody (p-PDGFRβ) that recognizes activated receptors [27]. The immunofluorescence for p-PDGFRβ in the dorsal horns of vehicle-treated rats remained at low levels, but was markedly increased 30 min after intrathecal PDGF-BB (10 pmol) administration (*P *< 0.001) (Figure 2A, B). By double-staining with cell type-specific markers, we found that almost all p-PDGFRβ-positive cells were double-labeled with OX-42 (a marker of microglia), but not with GFAP (an astrocyte marker), CC1 (an oligodendrocyte marker), MAP2 or NeuN (neuronal markers) (Figure 2C). These results indicate that activation of PDGFRβ evoked by intrathecally delivered PDGF-BB occurs specifically in microglia. Consistently, both PDGFRα and PDGFRβ mRNAs were detected in primary cultured microglia and in the spinal cord as well as positive control tissues (cerebral cortex and spleen) (Figure 2D). Furthermore, applying PDGF-BB (50 ng/ml) to primary cultured microglial cells enhanced the immunofluorescence for p-PDGFRβ (Figure 2E).

**Figure 3:**
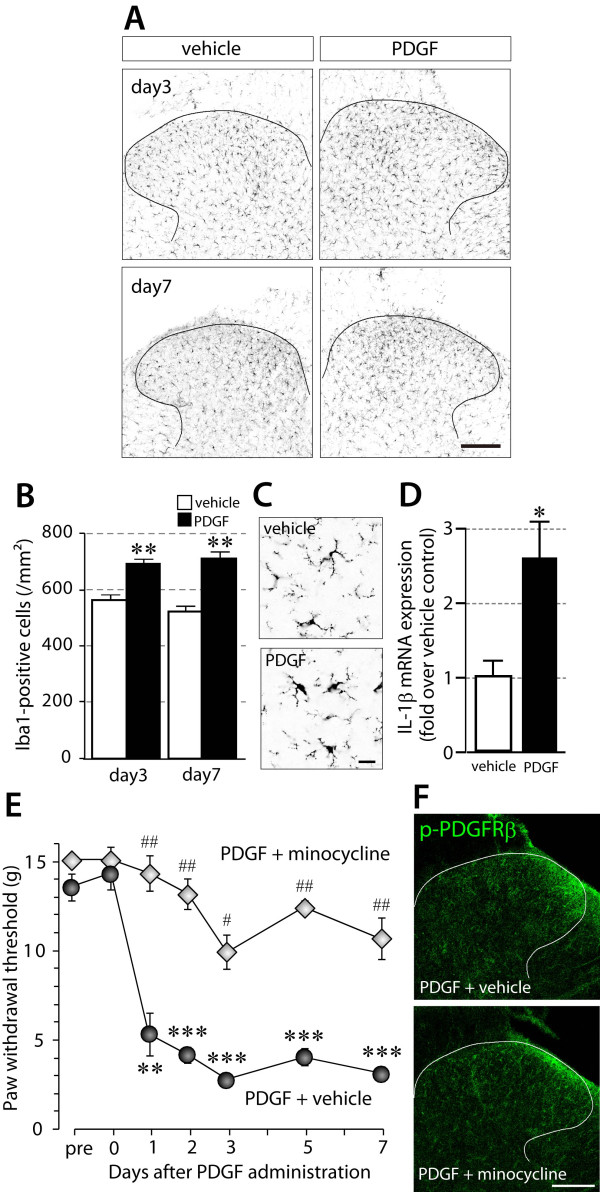
**Microglial activation is involved in PDGF-BB-induced tactile allodynia**. (A) The L5 spinal cord segments from PDGF-BB-administered rats at day 3 and 7 were subjected to immunohistochemistry using an anti-Iba1 antibody. Scale bar, 200 μm. (B) The number of Iba1-positive cells was counted in the dorsal horn. Data are means ± SEM of the cell number (day 3, n = 4; day 7, n = 3). **P < 0.01 vs vehicle by Student's *t*-test. (C) The magnified images of Iba1 staining at day 3. Scale bar, 20 μm. (D) Total RNA extracted from the L5 spinal dorsal horn 3 days after PDGF administration was subjected to quantitative analysis of interleukin-1β (IL-1β) mRNA expression by real-time RT-PCR. Data are means ± SEM of the fold change over vehicle control (n = 3). *P < 0.05 vs vehicle by Student's *t*-test. (E) The paw withdrawal thresholds of PDGF-BB (10 pmol)-administered rats were measured in a combined administration group with minocycline (100 μg, n = 4) or vehicle (PBS, n = 4). Minocycline or vehicle was intrathecally administered daily from one day before PDGF-BB administration. Data are means ± SEM of the thresholds. ***P < 0.001, **P < 0.01 vs before PDGF-BB administration; ^##^*P *< 0.01, ^#^*P *< 0.05 vs PDGF 10 pmol + vehicle group by Student's *t*-test. (F) Immunofluorescence for p-PDGFRβ in the L5 spinal dorsal horn 30 min after PDGF-BB (10 pmol) administration in minocycline- or vehicle-pre-administered rats. Minocycline or vehicle was intrathecally administered one day and 30 min before PDGF-BB administration. Scale bar, 200 μm.

Because the half life of PDGF *in vivo *is extremely short [28], it is predicted that PDGF-induced long-lasting tactile allodynia might be due to plastic changes in the spinal cord, especially in microglia. To investigate the status of microglia in the dorsal horn after PDGF stimulation, we performed immunohistochemical analysis using the microglia marker Iba1. After PDGF-BB (10 pmol) administration, the number of Iba1-positive cells in the dorsal horn was significantly increased on day 3 and day 7 compared with vehicle-treated controls (*P *< 0.01) (Figure 3A, B). Iba1-positive microglia in the dorsal horns of PDGF-BB-administered rats also showed an increase in Iba1 labeling and a tendency toward a hypertrophied morphology (Figure 3C). We also observed an increase in the level of expression of the proinflammatory cytokine interleukin-1β (IL-1β) (*P *< 0.05) (Figure 3D). Because these changes are consistent with the criteria for activated microglia *in vivo*, it is suggested that spinal microglia are activated by PDGF-BB.

To examine whether microglia are involved in PDGF-BB-induced tactile allodynia, we tested the effect of minocycline, which inhibits microglia activation [29,30], on the decrease in the paw withdrawal threshold after PDGF-BB administration. Daily intrathecal administration of minocycline (100 μg) from one day before PDGF-BB (10 pmol) administration significantly suppressed the decrease in paw withdrawal threshold (*P *< 0.05, day 3; *P *< 0.01, other testing days) (Figure 3E). This finding suggests that spinal microglia are involved in PDGF-BB-induced tactile allodynia. The mechanisms underlying the anti-allodynic effect of minocycline remains unclear, but we found that minocycline did not inhibit PDGF-induced PDGFRβ phosphorylation in the dorsal horn (Figure 3F), indicating that minocycline does not directly interrupt the PDGF binding to the PDGFRβ and PDGFRβ dimerization and autophosphorylation. Thus, it is conceivable that minocycline may produce its anti-allodynic effect through inhibiting the downstream consequences of PDGFRβ phosphorylation in microglia including p38 mitogen-activated protein kinase that is an important signaling molecule in tactile allodynia [11,15] and is also known as one of targets of minocycline [31,32].

**Figure 4:**
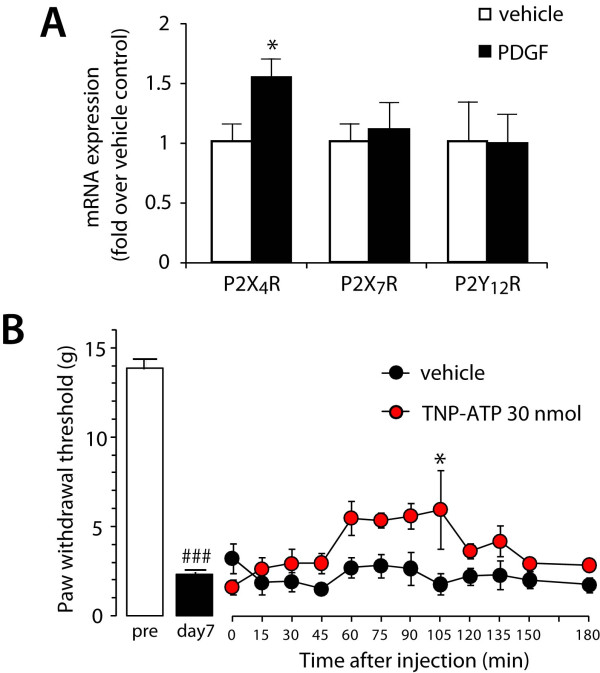
**ATP receptors relation to PDGF-BB-induced allodynia**. (A) Total RNA extracted from the L5 spinal cord on day 3 after PDGF-BB (10 pmol) administration was subjected to quantitative analysis of P2X_4_, P2X_7_, and P2Y_12 _receptors mRNA expression by real-time RT-PCR. Data are means ± SEM of the fold change over vehicle control (n = 5). *P < 0.01 vs vehicle by Student's *t*-test. (B) The paw withdrawal thresholds before (pre) and 7 days (day7) after intrathecal PDGF-BB (10 pmol) administration (n = 8). Then TNP-ATP (30 nmol) and vehicle (PBS) was intrathecally administered on day 7 and the changes in the paw withdrawal thresholds were measured (n = 4). Data are means ± SEM of the thresholds. ^###^*P *< 0.001 vs pre, *P < 0.05 vs vehicle group by Student's *t*-test.

Purinergic receptors expressed in microglia (P2X_4_, P2X_7 _and P2Y_12_) are implicated in neuropathic pain [12,13,33,34]. Thus, we examined the role of these receptors in PDGF-BB-induced allodynia. After intrathecal PDGF-BB (10 pmol) administration, the level of mRNA expression of P2X_4 _receptor in the spinal cord was significantly increased on day 3 (*P *< 0.05) (Figure 4A). By contrast, the mRNA levels of P2X_7 _and P2Y_12 _receptors were not changed. Furthermore, intrathecally administered TNP-ATP (30 nmol), an antagonist of P2X receptor subtypes P2X_1–4 _receptors, produced a significant attenuation of the decreased paw withdrawal threshold on day 7 after PDGF-BB (10 pmol) administration (*P *< 0.05) (Figure 4B). Considering that the anti-allodynic effect of TNP-ATP was weak, these results suggest that P2X_4 _receptors in the spinal cord are involved, at least in part, in the PDGF-BB-induced tactile allodynia.

**Figure 5:**
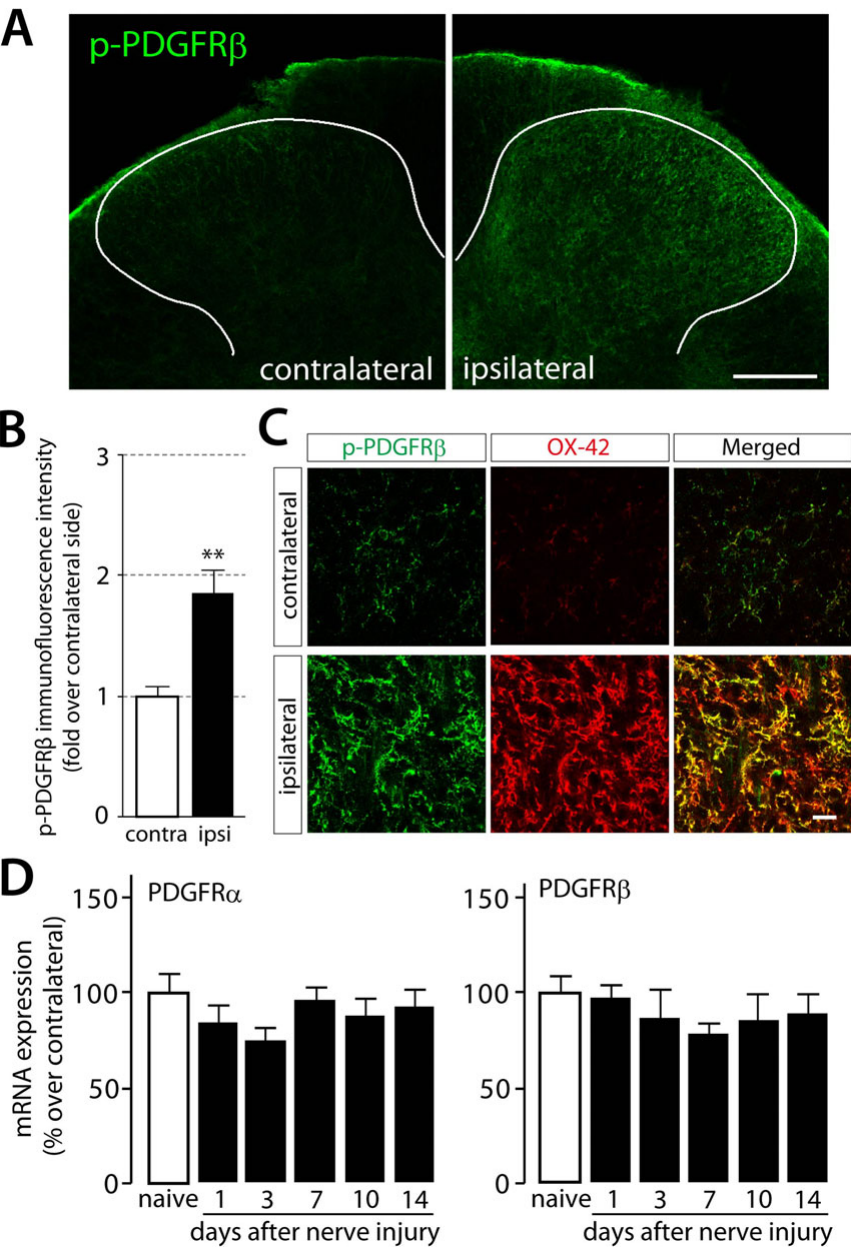
**Immunofluorescence of phosphorylated PDGF β-receptors and expression of PDGF receptor mRNAs in rats after nerve injury**. (A) The immunoreactivity for p-PDGFRβ was detected in the L5 spinal dorsal horn 4 days after nerve injury. Scale bar, 200 μm. (B) The intensity of p-PDGFRβ immunofluorescence was quantified in the dorsal horn region of contralateral side (contra) and ipsilateral side (ipsi) of nerve injured rats. Data represent the means ± SEM of the immunofluorescence intensity (n = 3). **P < 0.01 vs contra by Student's *t*-test. (C) Double immunofluorescence labeling of p-PDGFRβ (green) with OX-42 (red), a microglia marker. Scale bars, 20 μm. (D) Total RNA extracted from the L5 spinal cords of naive rats and peripheral nerve injured rats was subjected to quantitative analysis of PDGFR mRNA expression by real-time RT-PCR. Data are means ± SEM of the percentage over the naive value (ipsilateral side/contralateral side, n = 5).

Activation of PDGFRs in the spinal cord is implicated in tactile allodynia after peripheral nerve injury [24]. Thus, we determined the type of cells in which PDGFRβ activation occurs under a neuropathic pain condition. In contrast to the contralateral dorsal horn, where p-PDGFRβ immunofluorescence was low, we observed strong p-PDGFRβ immunofluorescence in the dorsal horn ipsilateral to the nerve injury; the level of p-PDGFRβ immunofluorescence in individual cells in this region was also much higher than that in individual cells in the dorsal horn contralateral to the nerve injury (*P *< 0.01) (Figure 5A, B). Furthermore, almost all p-PDGFRβ-positive cells were also labeled for the microglia marker OX-42 (Figure 5C). These results indicate that PDGFRβ activation in the dorsal horn occurs exclusively in microglia after nerve injury. How PDGFR activity is enhanced remains unclear, but we examined the time course for changes in the expression levels of PDGFR mRNAs after nerve injury and found no significant change during the period from 1 day to 14 days post-nerve injury (Figure 5D). It is thus possible that the enhanced PDGFR activity might be due to an increase in the level of endogenous PDGF within the dorsal horn after nerve injury, as suggested by a previous study [24]. Consistently, low levels of PDGFRβ phosphorylation in the dorsal horns of normal rats (Figure 2A) and in the contralateral dorsal horns of nerve-injured rats (Figure 5A) were observed. In the adult spinal cord, PDGF has been shown to be expressed in dorsal horn neurons [24]. It is thus assumed that PDGF might be a candidate for signaling molecules between neurons and microglia, thereby producing tactile allodynia, although further investigations are needed to determine the pattern and change in the expression of endogenous PDGFRβ ligands in the dorsal horn after nerve injury.

PDGFRs in the CNS have been previously reported to be expressed in O-2A progenitor cells, oligodendrocytes, and neurons [35-38]. In the present study, by showing that acute PDGF stimulation *in vivo *in adult rats induced PDGFRβ phosphorylation specifically in microglia, in addition to our results in *in vitro *experiments using cultured microglia, we provide the first evidence that microglia are the predominant cell type expressing functional PDGFRβs in the spinal cord. We further revealed that spinal microglia may mediate tactile allodynia caused by intrathecal administration of PDGF. Recently, Narita et al. [24] have shown that inhibiting PDGFR phosphorylation results in suppression of tactile allodynia after peripheral nerve injury, implying a crucial role for PDGF signaling in neuropathic pain. Notably, following peripheral nerve injury, a marked enhancement of PDGFRβ phosphorylation in dorsal horn microglia also occurred in a cell type-specific manner, indicating that spinal microglia may be crucial for PDGFR-mediated tactile allodynia under neuropathic pain conditions. It remains unknown how PDGF-stimulated microglia modulate pain processing in the dorsal horn, but we found an increase in the expression of IL-1β mRNA in the dorsal horn after PDGF administration. IL-1β enhances C-fiber-evoked responses in wide-dynamic-range dorsal horn neurons [39], enhances NMDA receptor-mediated Ca^2+ ^responses [40], and decreases GABA_A _receptor-mediated currents [41]. A recent study has also demonstrated a powerful role for this cytokine in excitatory and inhibitory synaptic transmission and an effect of this cytokine on neuronal activity in superficial dorsal horn neurons [42,43].

Therefore, IL-1β may be a candidate intermediary molecule between PDGF-stimulated microglia and dorsal horn neurons that contributes to central hypersensitization. Further investigation using microglia-specific IL-1β-knockout mice will clarify this issue.

## Methods

### Animals

Male Wistar rats (250–280 g, Japan SLC) were used. Rats were housed at a constant temperature of 23 ± 1°C with a 12 h light-dark cycle (light on 8:00 to 20:00) and fed food and water *ad libitum*. All of the animals used in the present study were obtained, housed, cared for, and used in accordance with the guidelines of Kyushu University.

### Microglia culture

Rat primary cultured microglia was prepared according to the method described previously [44]. In brief, the mixed glial culture was prepared from neonatal Wistar rats and maintained for 9–15 days in DMEM with 10% FBS. Microglia were obtained as floating cells over the mixed glial culture. The floating cells were collected by gentle shaking and transferred to culture dishes for each experiment.

### Drug administration

Under 2% isoflurane anesthesia, rats were implanted with a 32 gauge intrathecal catheter (ReCathCo, Allison Park, PA, USA) in the lumbar enlargement (close to L4-5 segments) for intrathecal drug administration. The catheter placement was verified by the observation of hindlimb paralysis induced by intrathecal administration of lidocaine (2%, 5 μl). Rats that failed to cause paralysis were excluded from the experiments. A recombinant human platelet-derived growth factor, PDGF-BB (0.1, 1 and 10 pmol/10 μl PBS; Millipore Bioscience Research Reagents, Temecula, California, USA), or PBS (10 μl, as a vehicle control) was intrathecally administered in naive rats. AG 17 [100 nmol/10 μl PBS containing dimethylsulfoxide (6%: final concentration); Calbiochem] or PBS containing 6% dimethylsulfoxide (10 μl, as a vehicle control) was intrathecally administered 30 min before PDGF-BB (10 pmol/10 μl PBS) administration. Minocycline (100 μg/10 μl PBS; Sigma) or PBS (10 μl, as a vehicle control) was intrathecally administered once a day from 1 day before PDGF-BB (10 pmol/10 μl PBS) administration. 2',3'-O-(2,4,6-trinitrophenyl)adenosine 5'-triphosphate, TNP-ATP (30 nmol/10 μl PBS; Sigma), or PBS (10 μl, as a vehicle control) was intrathecally administered on day 7 after PDGF-BB (10 pmol/10 μl PBS) administration.

### Neuropathic pain model and Behavioral tests

The left L5 spinal nerve of rats was tightly ligated with 5-0 silk suture and cut just distal to the ligature under 2% isoflurane anesthesia [12,45]. To assess the level of tactile allodynia, rats were placed individually in a wire mesh cage and habituated for 30–60 min to allow acclimatization to the new environment. From below the mesh floor, calibrated von Frey filaments (0.4–15 g; North Coast Medical, Morgan Hill, California, USA) were applied to the mid-plantar surface of the hindpaw. The 50% paw withdrawal threshold was determined using the up-down method [46].

### Immunohistochemistry

The rats used in the experiments were deeply anesthetized with pentobarbital (100 mg/kg, i.p.) and perfused transcardially with ice-cold PBS, followed by ice-cold 4% paraformaldehyde in PBS. The L5 segments of the lumber spinal cord were removed, post-fixed in the same fixative for 4 h at 4°C, and placed in 30% sucrose solution for 24 h at 4°C. Transverse spinal cord sections (30 μm) were sliced by a Leica CM 1850 cryostat and incubated in a blocking solution (3% normal goat serum) for 2 h at room temperature, and then incubated for 48 h at 4°C with the primary antibodies against phospho-PDGF β-receptor (rabbit polyclonal anti-phospho-Tyr1021 of PDGFRβ, 1:2000, Santa Cruz Biotechnology, Santa Cruz, CA, USA), or cell markers; microglia, OX-42 (mouse monoclonal anti-OX-42, 1:1000, Serotec, Oxford, UK) and ionized calcium-binding adapter molecule-1 (Iba1) (rabbit polyclonal anti-Iba1, 1:2000, Wako, Osaka, Japan); astrocytes, glial fibrillary acidic protein (GFAP) (mouse monoclonal anti-GFAP, 1:2000, Millipore Bioscience Research Reagents); oligodendrocytes, CC-1 (mouse monoclonal anti-APC, 1:500, Millipore Bioscience Research Reagents); neurons, neuronal nuclei (NeuN) (mouse monoclonal anti-NeuN, 1:200, Millipore Bioscience Research Reagents) and microtubule-associated protein-2 (MAP2) (mouse monoclonal anti-MAP2, 1:500, Millipore Bioscience Research Reagents). The sections were then washed and incubated for 3 h at room temperature with the fluorescent conjugated secondary antibodies (goat anti-rabbit IgG-conjugated Alexa Fluor 488 or goat anti-mouse IgG-conjugated Alexa Fluor 546, 1:1000, Invitrogen, Carlsbad, CA, USA). The sections were mounted with Vectashield (Vector Laboratories, Burlingame, CA, USA). Fluorescent images were obtained with a confocal microscope (LSM 5 Pascal; Carl Zeiss, Jena, Germany) and analyzed with Zeiss LSM Image Brower (Carl Zeiss). For quantitative assessment of the immunofluorescence staining, the spinal dorsal horn regions were outlined and the immunofluorescence intensity of the p-PDGFRβ was determined as the average pixel intensity within the field.

### Immunocytochemistry

Primary microglial cells were seeded on aminopropyltriethoxysilane-coated glass (Matsunami, Osaka, Japan) at 5 × 10^4 ^cells/well and incubated for 1 h. After the culture media were replaced with serum-free media, cells were incubated for 2 h and subsequently treated with PBS as a control or 50 ng/ml PDGF-BB for 10 min [47], and then fixed in 3.7% formaldehyde in PBS for 30 min at 25°C. The cells were permeabilized and blocked by incubating them with blocking solution (3% normal goat serum and 0.3% Triton X-100 in PBS) for 15 min at 25°C, and then incubated overnight at 4°C with the primary antibodies against phospho-PDGF β-receptor (1:400) and OX-42 (1:1000). After washing, the cells were incubated for 1 h with appropriate fluorescent-conjugated secondary antibodies (goat anti-rabbit IgG-conjugated Alexa Fluor 488 or goat anti-mouse IgG-conjugated Alexa Fluor 546, 1:1000) and coverslipped in Vectashield containing 4',6-diamidino-2-phenylindole (DAPI) (Vector Laboratories, Burlingame, CA, USA). Fluorescent images were obtained and analyzed as mentioned above.

### Real-Time Quantitative RT-PCR

The rats used in the experiments were deeply anesthetized with pentobarbital (100 mg/kg, i.p.) and perfused transcardially with ice-cold PBS. The L5 segments of lumber spinal cord were removed immediately and were subjected to total RNA extraction using Trisure (Bioline, Danwon-Gu, South Korea) according to the protocol of the manufacturer and purified with RNeasy mini plus kit (Qiagen, Valencia, CA, USA). The amount of total RNA was quantified by measuring OD_260 _using a Nanodrop spectrophotometer (Nanodrop, Wilmington, DE, USA). For reverse transcription with random 6-mer primers, 100 ng of total RNA was transferred to the reaction with Prime Script reverse transcriptase (Takara, Kyoto, Japan). Quantitative PCR was performed with Premix Ex *Taq *(Takara) using a 7500 real-time PCR system (Applied Biosystems, Foster City, CA, USA) according to protocol of the manufacturer, and the data were analyzed by 7500 System SDS Software 1.3.1 (Applied Biosystems) using the standard curve method. Expression levels were normalized to the values for glyceraldehyde-3-phosphate dehydrogenase (GAPDH). The TaqMan probes and primers for interleukin-1β (IL-1β) (Taqman probe, 5'-FAM-TTCTCCACCTCAATGGACAGAACATAAGCCA-TAMRA-3'; forward primer, AAATGCCTCGTGCTGTCTGA; reverse primer, GTCGTTGCTTGTCTCTCCTTGTAC), P2X_4 _receptor (P2X_4_R) (Taqman probe, 5'-FAM-AGGAGGAAAACTCCCTCTTCATCATGACCA-TAMRA-3'; forward primer, TGGCGGACTATGTGATTCCA; reverse primer, GGTTCACGGTGACGATCATG), P2X_7 _receptor (P2X_7_R) (Taqman probe, 5'-FAM-AAAGCCTTCGGCGTGCGTTTTGA-TAMRA-3'; forward primer, CATGGAAAAGCGGACATTGA; reverse primer, CCAGTGCCAAAAACCAGGAT), P2Y_12 _receptor (P2Y_12_R) (Taqman probe, 5'-FAM-CACCAGACCATTTAAAACTTCCAGCCCC-TAMRA-3'; forward primer, TAACCATTGACCGATACCTGAAGA; reverse primer, TTCGCACCCAAAAGATTGC), PDGF receptor α-subtype (PDGFRα) (Taqman probe, 5'-FAM-ATATTCTCCCTTGGTGGCACACCCTACC-TAMRA-3'; forward primer, ACGTCTGGTCTTATGGCGTTCT; reverse primer, CATCCTGTATCCGCTCTTGATCT), and PDGFRβ (Taqman probe, 5'-FAM-AACGACTCACCAGTGCTCAGCTACACAGAC-TAMRA-3'; forward primer, GTCCCATCTGCCCCTGAAA; reverse primer, GGTCTCGGTGAACACAGTTCTTAG), as well as the probe and primer for GAPDH, were obtained from Applied Biosystems.

### Statistical Analysis

All data are presented as means ± SEM. The statistical analyses of the results were evaluated by using the Student's *t *test or two-way repeated measures ANOVA with Bonferroni post tests.

## Competing interests

The authors declare that they have no competing interests.

## Authors' contributions

JM performed the majority of experiments, analyzed the data, and drafted the manuscript; MT designed and supervised the experiments, and wrote the manuscript; HST. supervised some experiments; KI coordinated the project, supervised the experiments, helped to interpret the data, edited the manuscript. All authors discussed the results and commented on the manuscript.
